# Preoperative Vitamin D and Calcium Administration in Patients Undergoing Thyroidectomy: A Systematic Review and Meta‐analysis of Randomized Controlled Trials

**DOI:** 10.1002/oto2.116

**Published:** 2024-02-16

**Authors:** Mohammed Alhakami, Ghassan B. Lajdam, Abdullah A. Ghaddaf, Sarah Alayoubi, Shaden Alhelali, Mohammad Alshareef, Jabir Alharbi

**Affiliations:** ^1^ College of Medicine King Saud bin Abdulaziz University for Health Sciences Jeddah Saudi Arabia; ^2^ King Abdullah International Medical Research Center Jeddah Saudi Arabia; ^3^ Department of Otolaryngology‐Head & Neck Surgery, King Saud bin Abdulaziz University for Health Sciences King Abdulaziz Medical City Jeddah Saudi Arabia; ^4^ Collage of Medicine Ibn Sina National Collage for Medical Studies Jeddah Saudi Arabia; ^5^ Head & Neck and Skull Base Health Center King Abdullah Medical City Makkah Saudi Arabia

**Keywords:** calcium, hypocalcemia, thyroid disease, thyroidectomy, vitamin D

## Abstract

**Objective:**

This systematic review and meta‐analysis aimed to assess whether preoperative administration of calcium and vitamin D prevents postoperative hypocalcemia.

**Data Sources:**

A computerized search in Medline, Embase, and CENTRAL databases was performed.

**Review Methods:**

Trials comparing preoperative calcium and vitamin D administration with either placebo or nothing were eligible for inclusion. The primary outcomes were the occurrence of laboratory hypocalcemia, mean postoperative calcium level, and symptomatic hypocalcemia. The secondary outcomes were the development of permanent hypoparathyroidism and length of hospitalization. Continuous outcomes were represented as standardized mean difference (SMD), and dichotomous outcomes were represented as risk ratio (RR).

**Results:**

Nine trials that enrolled 1079 patients were found eligible. Postoperative laboratory hypocalcemia occurred less in patients who received preoperative calcium and vitamin D, but it was not statistically significant (RR = 0.77, 95% CI: 0.60‐1.00; *P* = .05). Mean postoperative calcium level was significantly higher in the intervention group (SMD = 0.10, 95% CI: 0.07‐0.12; *P* < .00001). The number of patients with symptomatic hypocalcemia was significantly lower in the intervention group (RR = 0.54, 95% CI: 0.38‐0.76; *P* = .0005). There was no significant difference between the 2 groups in cases of permanent hypoparathyroidism and length of hospitalization.

**Conclusion:**

Administration of calcium and vitamin D preoperatively achieves lower rates of postthyroidectomy symptomatic hypocalcemia in comparison with no intervention.

Hypocalcemia is the most common complication following thyroidectomy.^1^ In the literature, it was reported that the overall incidence of postthyroidectomy hypocalcemia ranges from 19% to 38%.^2^ In a recent study, 5.8% of postthyroidectomy patients developed severe hypocalcemia, and 83.2% required intravenous (IV) calcium therapy.^3^ Additionally, the risk of mortality and morbidity increases with severe hypocalcemia.^4^ Postthyroidectomy hypocalcemia can be either symptomatic or asymptomatic. Paresthesia, numbness, and tingling sensations are the common neurological symptoms to appear. Cardiac manifestations are also observed and include irregular pulse, palpitations, and syncope.^5^ The mainstay preventive measure of postthyroidectomy hypocalcemia is avoiding injury to the parathyroid (PTH) gland during surgery.^6^, ^7^, ^8^ However, the injury is sometimes inevitable, and there is a need to investigate other preventative options. In recent systematic reviews by Khatiwada et al and Casey et al, the use of preoperative calcium and vitamin D supplements showed a significant decrease in rates of postthyroidectomy laboratory and symptomatic hypocalcemia. However, these reviews were limited by the nonrandomized and qualitative nature of their studies.^9^, ^10^ Another meta‐analysis found a significant reduction in symptomatic hypocalcemia. The study was limited, however, by the small number of included randomized controlled trials (RCTs) and the small sample size.^11^ Recently, numerous RCTs on the use of preoperative calcium, vitamin D, or both to prevent postthyroidectomy hypocalcemia have been published. Some studies showed supplements were not effective to prevent postoperative hypocalcemia,^12^, ^13^ whereas others showed a significant protective effect.^14^, ^15^ Hence, we conducted this systematic review and meta‐analysis to assess the efficacy of preoperative calcium and vitamin D administration for prevention of postthyroidectomy laboratory and symptomatic hypocalcemia.

## Methods

We reported this systematic review and meta‐analysis according to the Preferred Reporting Items for Systematic Reviews and Meta‐analysis (PRISMA) checklist.^16^ This study was based on a pre‐specified protocol registered in PROSPERO (CRD42022356363). This study was waived by the International Review Board because it does not include human subjects and only uses pre‐published data.

### Information Sources and Search Strategy

A computerized search was performed in the following databases: MEDLINE, Embase, Cochrane Central register of Controlled Trials (CENTRAL), and Google Scholar. The last systemic search was on November 15, 2022, with no restrictions on the date. Additionally, scanning reference lists was performed. The following terms: thyroidectomy, thyroid gland, thyroid disease, vitamin D, calcitriol, alfacalcidol, and calcium, were used in our search strategy. The complete search strategy is provided in Supplemental Table S1, available online.

### Inclusion and Exclusion Criteria

Adults who were at least 18 years old and underwent a near‐total or total thyroidectomy were eligible for inclusion. Studies on individuals who had lobectomy or hemithyroidectomy were excluded. We included studies in which participants received vitamin D with or without calcium supplementation prior to the operation. Studies that only included a post‐operative or routine calcium supplement for thyroidectomy patients were excluded.

Eligible trials must have at least one of the following prespecified outcomes: postoperative mean calcium level, number of patients with laboratory hypocalcemia, number of patients with symptomatic hypocalcemia, and number of patients with permanent hypoparathyroidism. We only included RCTs. Animal studies, observational studies, systematic reviews, case reports, opinion articles, and conference abstracts were all excluded. Studies written in languages other than English and unavailable full texts were also excluded. Study eligibility screening was carried out by 2 reviewers independently and in duplicate. First, the reviewers carefully screened the titles and abstracts. Eligible abstracts were then assessed by full text assessment. Disagreements were settled through discussion or a third reviewer.

### Validity and Quality Assessment

Validity assessment has been done independently and in duplicate by 2 reviewers using revised Cochrane Risk of Bias Tool 2.^17^ Any disagreement was settled by discussion or the opinion of a third reviewer.

### Data Extraction

Two reviewers independently extracted the data in duplicate using a predefined data collection model. We gathered data on participants' demographics, indication for surgery, operation type, PTH re‐implantation, mean preoperative calcium level, the dose of intervention, duration of administration, duration of follow‐up, mean postoperative calcium level, laboratory hypocalcemia, symptomatic hypocalcemia, permanent hypoparathyroidism, and length of hospital stay.

### Data and Meta‐analysis

The meta‐analysis was performed using the random‐effects model in RevMan (Review Manager) version 5.3 (Cochrane Collaboration). The confidence level was set at 95%, with a threshold of *P* < .05. We used I2 and the *P*‐value of Chi^2^ to assess the statistical heterogeneity. All outcomes were pooled by the inverse variance weighting methods. A subgroup analysis was performed based on the type of intervention (ie, calcium and vitamin D, vitamin D only) and the mean calcium levels on postoperative day 1 (POD 1), postoperative day 2 (POD 2), and postoperative day 3 (POD 3). Also, the continuous outcomes, mean postoperative calcium level, and length of hospitalization were represented as standardized mean difference (SMD) whereas the dichotomous outcomes, laboratory hypocalcemia, symptomatic hypocalcemia, and permanent hypoparathyroidism were represented as risk ratios (RRs). Finally, The Grading of Recommendation Assessment, Development and Evaluation (GRADE) was utilized to determine the degree of certainty in the evidence for each outcome.

## Results

### Study Selection

**Figure 1:**
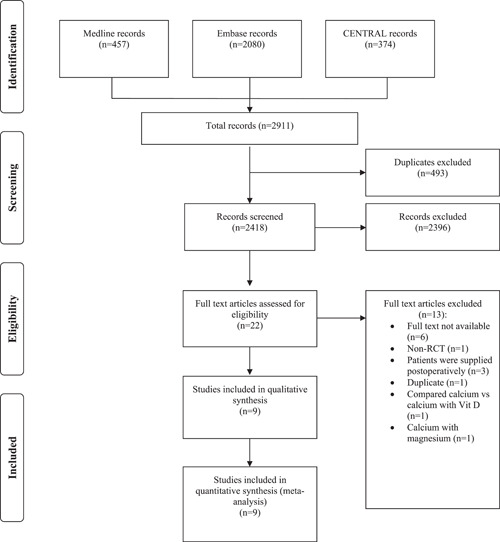
PRISMA flow diagram of study selection.

Figure 1 demonstrates the flowchart of the included RCTs. The computerized and manual search yielded 2911 records. After removing duplicates, a number of 2396 articles were further screened, and 22 were eligible for full‐text assessment. Eventually, 9 articles were deemed eligible.

### Study Characteristics

This review included 1079 participants, divided almost evenly among the intervention and control groups. The lowest mean age of the participants was 23.79 years old, and the highest was 59.34 years old. Most participants were female (76.74%). Five of the RCTs used vitamin D only as a preoperative supplement, and the other 4 RCTs used calcium and vitamin D preoperatively. There were different forms of vitamin D across the study such as alfacalcidol, calcitriol, ergocalciferol, and others that were specified in Table 1. The duration of dose administration ranged from 1 day prior to the surgery to 6 weeks prior to the surgery. Donahue et al had the shortest follow‐up duration (ie, 3 days), while Genser et al and Rowe et al had the longest (ie, 6 months).^12^, ^13^, ^15^ Further details on study characteristics are shown in Table 1.

**Table 1:** Characteristics of the Included Studies

		Number of participants		Mean age (in years)							Outcomes measured	
Study, year	Type of intervention	Intervention	Placebo or no intervention	Intervention	Placebo or no intervention	Gender (number of females)	Dose of intervention	Route/form of vitamin D	Duration of administration	Follow‐up	Subjective outcomes	Objective outcomes
Genser 2014	Vitamin D	111	108	53.3 ± 13	52.9 ± 14	174	Vitamin D: 2 mg	Oral/alfacalcidol	A day before surgery to postoperative day 8	6 months	Symptomatic hypocalcemia	MCL, POHC, LOH
Jaan 2017	Calcium + Vitamin D	30	30	36.43 ± 11.54	38.13 ± 15.71	46	Calcium: 500 mg every 6 h Vitamin D: 0.25 µg every 6 h	Oral/calcitriol	7 days before and 7 days after the surgery	1 month	Symptomatic hypocalcemia	MCL, POHC
Rowe 2018	Vitamin D	72	78	49 (15)^a^	55 (17)^a^	114	Vitamin D: 50,000 IU 6 tablets (300,000)	Oral/cholecalciferol	7 days prior to thyroidectomy	6 months	Symptomatic hypocalcemia	MCL, POHC, LOH, permanent hypoparathyroidism
Malik 2019	Calcium + Vitamin D	46	46	38.673 ± 8.63	41.217 ± 9.52	43	Vitamin D: 2,00,000 IU + Calcium: 1 g	Oral/not specified	24 h preoperatively	NR	NR	POHC
Ramouz 2020	Vitamin D	50	50	44 ± 1.37	41.48 ± 1.60	91	Vitamin D: 50,000 IU	Oral/ergocalciferol	Once a week for 4 weeks preoperatively	2 weeks	Symptomatic hypocalcemia	MCL, POHC
Shonka 2021	Vitamin D	23	24	49.9 (14.5)^a^	46.0 (16.5)^a^	40	Vitamin D: 1 μg	Oral/calcitriol	2× a day for 1 week preceding thyroidectomy	30 days	Symptomatic hypocalcemia	MCL, POHC, LOH
Donahue 2021	Calcium + Vitamin D	38	44	59.34 (15.74)^a^	52.53 (13.27)^a^	59	Calcium: 1500 mg PO TID + Vitamin D: 0.25 mg PO BID	Oral/calcitriol	Starting 5 days before operation	3 days	Symptomatic hypocalcemia	MCL, POHC, LOH
Sonnenberg 2021	Vitamin D	130	116	48.5 (50.45; 46.5)^b^	48.9(51.2;46.6)^b^	185	Vitamin D: 0.5 μm b.i.d	Oral/calcitriol	Three days preoperatively	30 days	post‐operative quality of life	POHC, LOH, duration of recovery of biochemical normocalcemia
Sasi 2022	Calcium + Vitamin D	41	42	39.74 ± 12.79	23.79 ± 4.33	76	Calcium carbonate 2000 mg/day + cholecalciferol 60,000 units every week	Oral/cholecalciferol	6 weeks before surgery	1 month	Symptomatic hypocalcemia	POHC

Abbreviations: BID, twice a day; LOH, length of hospital stay; MCL, mean calcium level; NR, not reported; POHC, postoperative hypocalcemia; TID, three times a day.

^a^ Data are mean (SD).

^b^ Mean and 95% confidence interval.

### Validity Assessment

**Figure 2:**
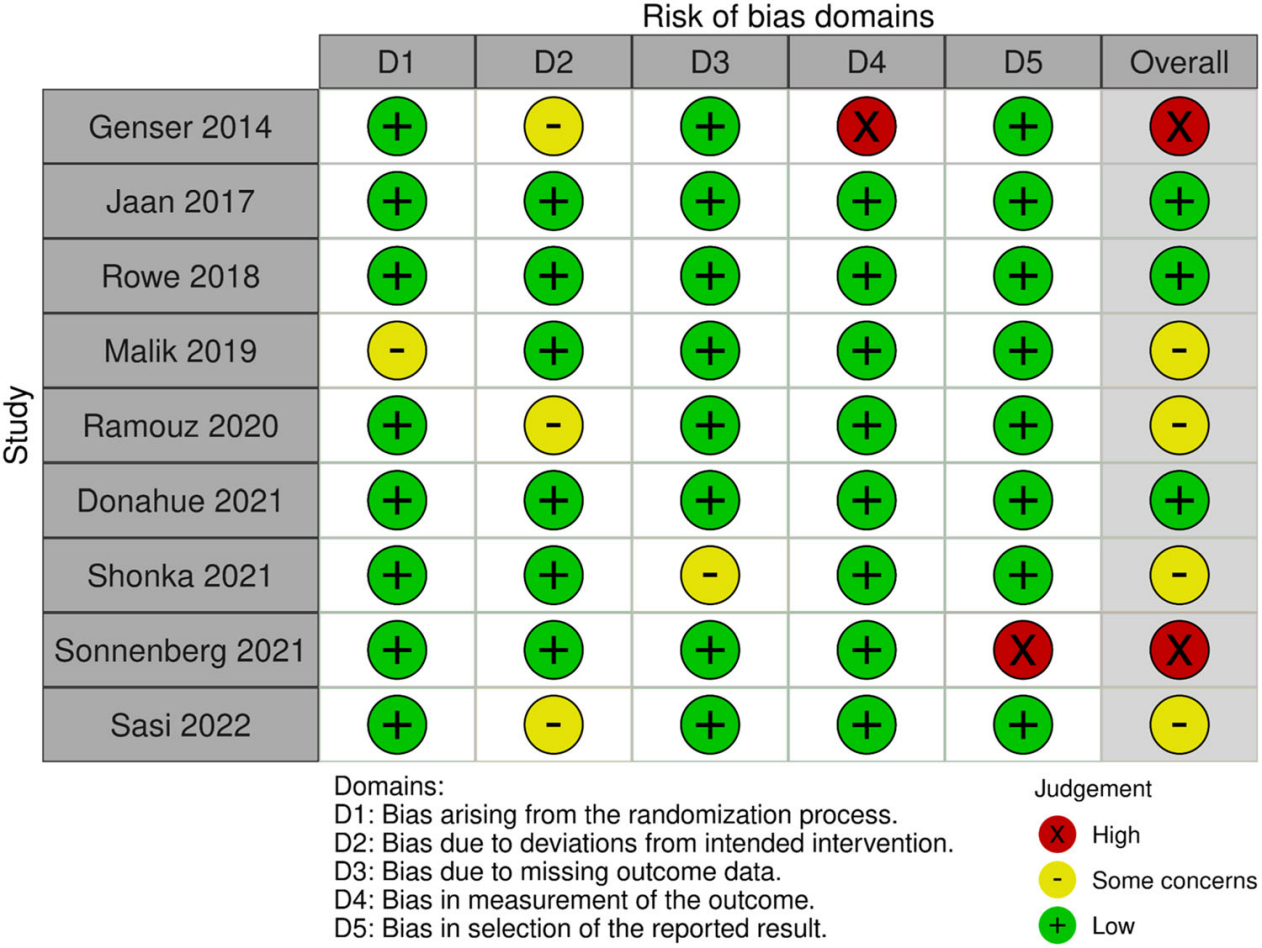
“Risk of bias summary” review authors' judgements about each risk of bias item for each included study.

**Figure 3:**
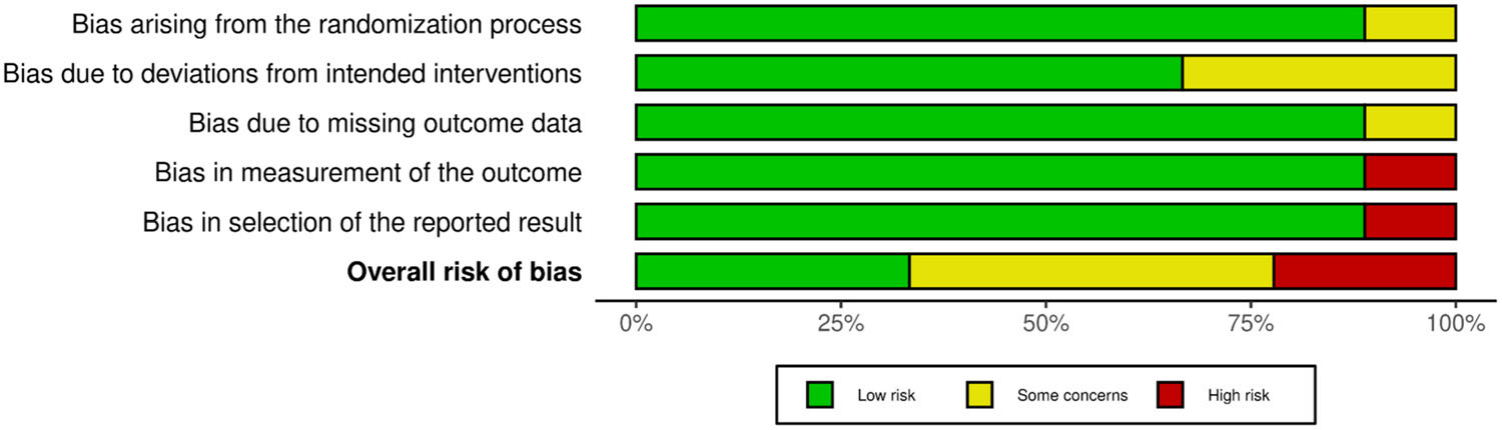
“Risk of bias graph” review authors' judgements about each risk of bias item presented as percentages across all included studies.

Out of the 9 trials, 4 had overall some concerns, 3 trials had low risk of bias, and 2 trials had high risk of bias. The details of the risk of bias domains are shown in Figures 2 and 3.

### Laboratory Postoperative Hypocalcemia

**Figure 4:**
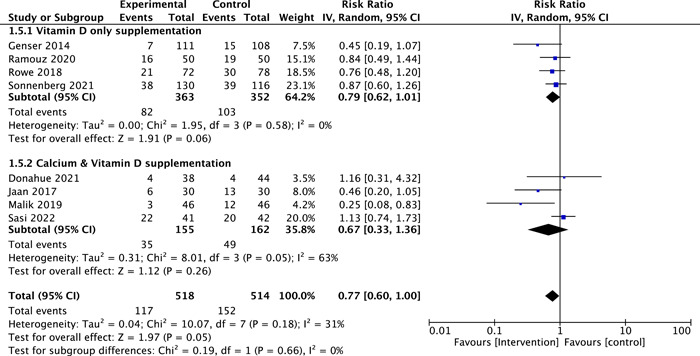
Forest plot of laboratory post‐operative hypocalcemia.

Eight studies have reported laboratory postoperative hypocalcemia (n = 1032).^12^, ^13^, ^14^, ^15^, ^18^, ^19^, ^20^, ^21^ Participants who received calcium and vitamin D, or vitamin D only had a lower occurrence of laboratory hypocalcemia than the control group. However, this finding was not statistically significant (RR = 0.77, 95% confidence interval [CI]: 0.60‐1.00; *P* = .05, *I* ^2^ = 31%, high‐certainty evidence, Figure 4). The subgroup analysis based on the type of intervention, showed no significant difference between the combined supplement (ie, vitamin D and calcium) and vitamin D only (*P* = .66, Figure 4).

### Symptomatic Hypocalcemia

**Figure 5:**
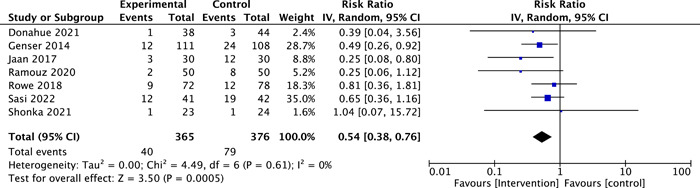
Forest plot of symptomatic postoperative hypocalcemia.

Seven RCTs reported the number of patients with symptomatic hypocalcemia (n = 741).^12^, ^13^, ^14^, ^15^, ^19^, ^21^, ^22^ In the intervention group, the percentage of symptomatic participants was 10.96%. The control group, on the other hand, showed a higher incidence of symptomatic hypocalcemia (ie, 21%). The pooled effect estimates of symptomatic postoperative hypocalcemia showed a significant difference favoring the intervention group (RR = 0.54, 95% CI: 0.38‐0.76; *P* = .0005, *I* ^2^ = 0%, moderate‐certainty evidence, Figure 5).

### Mean Postoperative Calcium Level

**Figure 6:**
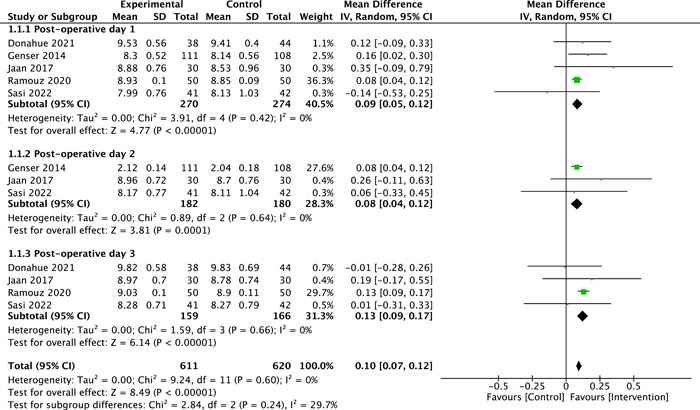
Forest plot of mean post‐operative calcium levels.

The mean postoperative calcium level was reported by 5 RCTs (n = 544).^13^, ^14^, ^15^, ^19^, ^21^ Overall, mean postoperative calcium levels were significantly higher in the intervention group in comparison to control group (SMD = 0.10, 95% CI: 0.07‐0.12; *P* < .00001, *I* ^2^ = 0%, Figure 6). The subgroup analysis was conducted based on calcium levels on POD 1, POD 2, and POD 3. The pooled effect estimate revealed a significantly higher level of calcium in intervention group compared to control group at POD 1 (SMD = 0.09, 95% CI: 0.05‐0.12; *P* < .00001, *I* ^2^ = 0%, Figure 6), POD 2 (SMD = 0.08, 95% CI: 0.04‐0.12; *P* = .0001, *I* ^2^ = 0%, Figure 6), and POD 3 (SMD = 0.13, 95% CI: 0.09‐0.17; *P* < .00001, *I* ^2^ = 0%, moderate‐certainty evidence, Figure 6).

### Permanent Hypoparathyroidism

**Figure 7:**
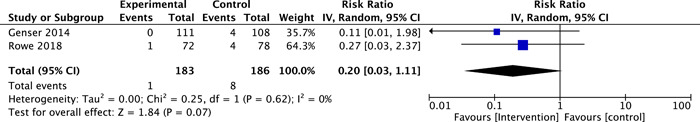
Forest plot of permanent hypoparathyroidism.

Two RCTs investigated the incidence of permanent hypoparathyroidism (n = 269).^12^, ^15^ In total, only 1 patient in the intervention group was diagnosed with permanent hypoparathyroidism, whereas 8 patients developed this complication in the control group. However, there was no statistically significant difference between the 2 groups (RR = 0.20, 95% CI: 0.03‐1.11; *P* = .07, *I* ^2^ = 0%, low‐certainty evidence, Figure 7).

### Length of Hospital Stay

**Figure 8:**
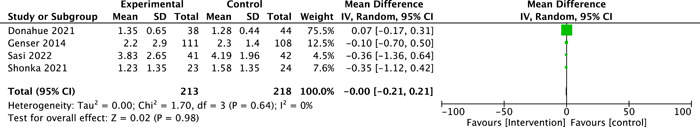
Forest plot of the length of hospital stay in days.

A total of 4 RCTs reported the length of hospital stay (n = 431).^13^, ^15^, ^21^, ^22^ The pooled effect showed no significant difference between the 2 groups (SMD = −0,00, 95% CI: −0.21‐0.21; *P* = .98, *I* ^2^ = 0%, low‐certainty evidence, Figure 8).

## Discussion

This systematic review and meta‐analysis included 9 RCTs that compared preoperative supplements (ie, calcium and vitamin D, both) with placebo or no intervention in adults who underwent thyroidectomy. The pooled estimates were in favor of the intervention group for 3 outcomes: laboratory hypocalcemia, mean calcium level, and symptomatic hypocalcemia. However, no significant differences were detected between the 2 groups in terms of the incidence of permanent hypoparathyroidism and length of hospitalization.

### Postoperative Laboratory and Symptomatic Hypocalcemia

Our results showed a significantly lower number of patients in terms of postoperative symptomatic hypocalcemia in the intervention group compared to the control group. However, laboratory hypocalcemia showed insignificant results. Our findings can be explained by the intrinsic role of vitamin D and the duration it takes to exert its effects on calcium levels. Vitamin D has a principal role in regulating calcium intestinal absorption and both bone and renal resorption.^23^ However, vitamin D generally takes up to 2 weeks to exert its effect on serum calcium levels.^24^, ^25^ Therefore, preoperative administration of vitamin D is thought to be essential in terms of giving sufficient time to increase calcium absorption from the gut. Furthermore, our results for symptomatic hypocalcemia were consistent with those of Sittitrai et al and Maxwell et al, both of which were randomized and nonrandomized studies.^26^, ^27^ However, their results on the laboratory hypocalcemia outcome were contradictory to our findings.^26^, ^27^ The literature is not in complete agreement as there are other studies that found opposing results. For instance, a retrospective cohort study found no statistically significant lower rates of symptomatic hypocalcemia.^28^ Another controlled trial found that postoperative calcium and vitamin D administration did not reduce the risk of postoperative hypocalcemia.^29^ These inconsistent results may be due to several factors, including differences in study design, sample size, duration of administration, and duration of follow‐up.

### Permanent Hypoparathyroidism

As for permanent hypoparathyroidism, our analysis showed no significant differences between the intervention and the control group. The definition of permanent hypoparathyroidism varies among the guidelines. However, the clinical decision is taken according to the following domains: biochemical PTH level, clinical features of hypoparathyroidism (ie, acute as hypocalcemia symptoms or chronic as extrapyramidal symptoms), and the chronicity of the condition after thyroidectomy.^30^, ^31^, ^32^, ^33^, ^34^, ^35^ According to a previous study, the incidence of permanent hypoparathyroidism, defined as PTH < 10 pg/mL at 1‐year postthyroidectomy, was 1.9%. Similar to our results, Kannan et al showed lower rates of permanent hypoparathyroidism in the intervention group compared to the control group, but there was no statistically significant difference.^36^ However, we could not find studies in the literature that found opposing results. Therefore, we believe that preoperative calcium and vitamin D supply has no clinical significance on permanent hypoparathyroidism.

### Length of Hospital Stay

Although our review reported lower number of days of hospitalization in the intervention group, the difference was not statistically significant. On contrary, Maxwell et al found that the length of hospitalization was significantly shorter for patients who received preoperative calcium and vitamin D.^27^ There are many factors, other than the surgery, that can play a role in the length of hospitalization such as para‐clinical services, consultation requests, doctor rounds, gender, type of insurance, and discharge delay time.^37^ We believe that the data are not yet inclusive, and more research should discuss this outcome based on factors affecting length of hospitalization.

### Strengths and Limitations

This systematic review and meta‐analysis provide inclusive evidence from RCTs on this topic that were not previously reviewed. Second, we had a relatively larger sample size compared to the previously published reviews.^11^ Third, this review adopted a stricter inclusion criterion as we only included preoperative prophylaxis compared to other reviews that evaluated both preoperative and postoperative prophylaxis.^27^, ^38^ We acknowledge that there are some limitations in our review. First, we were limited by the quality of evidence, as most studies were judged to have some concerns and high risk of bias. Second, the number of trials for some outcomes, such as permanent hypoparathyroidism, was low. Third, some studies reported a short period of follow‐up (ie, 3 days, 2 weeks, or 1 month). Fourth, excluding non‐English articles may limit the inclusivity and generalizability of the findings. Finally, we did not conduct a subgroup analysis of patients at high risk of developing postoperative hypocalcemia (ie, substernal multinodular goiter, central neck dissection, thyroid malignancy).^39^

## Conclusion

This systematic review and meta‐analysis showed lower rates of postthyroidectomy symptomatic hypocalcemia in patients who received calcium and vitamin D compared to patients who received a placebo or no intervention. There was no difference between both groups in terms of laboratory hypocalcemia, the length of hospital stay, and the incidence of permanent hypoparathyroidism. Prethyroidectomy administration of vitamin D and calcium can benefit patients. However, the impact and benefit are only in symptoms, and there is not any difference in terms of other outcomes. Furthermore, since the laboratory result was near enough to be considered significant, it can be further investigated with a larger sample size and a longer duration of calcium and vitamin D administration. Also, future studies should have a longer follow‐up period to assess the long‐term impact of this intervention.

Overall, it could be recommended to reduce the incidence of symptomatic hypocalcemia and improve the quality of life of thyroidectomy patients but, due to the limitations of the results, we advise caution in adopting this finding.

## Author Contributions

**Mohammed Alhakami**, participated in screening, data analysis, writing and reviewing the manuscript; **Ghassan B. Lajdam**, protocol synthesis, data collection, risk of bias, and editing the manuscript. **Abdullah A. Ghaddaf**, protocol synthesis, data analysis, and editing manuscript. **Sarah Alayoubi**, screening, data collection, and editing the manuscript; **Shaden Alhelali**, screening, data collection, and editing the manuscript; **Mohammad Alshareef**, conception the work, revising the work critically; final approval of the version; agreed to be accountable for work in ensuring that questions related to the accuracy or integrity are investigated; **Jabir Alharbi**, conception the work, revising the work critically; final approval of the version; agreed to be accountable for work in ensuring that questions related to the accuracy or integrity are investigated.

## Disclosures

### Competing interests

None.

### Funding source

None.

## Supporting information

Supplementary table 1: Search strategy.
