# Supplementary material for: Preoperative Vitamin D and Calcium Administration in Patients Undergoing Thyroidectomy: A Systematic Review and Meta‐analysis of Randomized Controlled Trials
Source: OTO Open. 2024 Feb 16;8(1):e116. doi: 10.1002/oto2.116 (PMC10870329; doi:10.1002/oto2.116)
Supplement: Supplementary file 1 — Supplementary table 1: Search strategy. [file OTO2-8-e116-s001.pdf]

Search strategy for Medline, Embase, and CENTRAL last performed on November 15, 2022 (**n=2418**):

1. exp Thyroidectomy/
2. Thyroidectomy$.mp.
3. exp Thyroid Gland/
4. Thyroid Gland$.mp.
5. exp Thyroid Diseases/ or exp Thyroid Neoplasms/
6. Thyroid$.mp.
7. Thyroid lobectomy$.mp.
8. 1 or 2 or 3 or 4 or 5 or 6 or 7
9. exp Vitamin D/
10. Vitamin D$.mp.
11. exp Receptors, Calcitriol/ or exp Calcitriol/
12. Calcitriol$.mp.
13. Alfacalcidol$.mp.
14. exp Ergocalciferols/
15. Ergocalciferols$.mp.
16. exp Cholecalciferol/
17. Cholecalciferol$.mp.
18. exp Ergosterol/
19. Ergosterol$.mp.
20. exp Calcium/
21. Calcium$.mp.

22. 9 or 10 or 11 or 12 or 13 or 14 or 15 or 16 or 17 or 18 or 19 or 20 or 21

23. exp Controlled Clinical Trial/ or exp Clinical Trial/ or exp Randomized Controlled Trial/

24. Trial$.mp.

25. 23 or 24

26. 8 and 22 and 25
